# Mini open triple tunnel- double flip button techniques in treatment of acute acromioclavicular joint injuries: Case report

**DOI:** 10.1016/j.tcr.2021.100450

**Published:** 2021-02-26

**Authors:** Romy Deviandri, I.G.M. Febry Siswanto, Andri M.T. Lubis

**Affiliations:** a Department of Orthopedics, University of Groningen, University Medical Center Groningen, Groningen, the Netherlands; b Department of Physiology, Faculty of Medicine, Universitas Riau, Arifin Achmad Hospital, Pekanbaru, Indonesia; c Department of Orthopedics Sport Injury, Royal Progress Hospital Jakarta, Indonesia; d Department of Orthopedics, Faculty of Medicine, Universitas Indonesia, Cipto Mangunkusumo Hospital, Jakarta, Indonesia

**Keywords:** Triple tunnel, Double button, V-loop suture, TTDB, AC joint injuries

## Abstract

Acute acromioclavicular (AC) joint injuries are common and often occur in a contact sport activity. Most acute AC joint injuries surgery techniques focus on coracoclavicular (CC) ligament complex fixation; by single or double clavicle tunnel, but persisting vertical instability. In this paper, we introduce mini open triple tunnel- double flip button (TTDB) technique for acute AC joint dislocation by adding tunnel on clavicle to expand coverage of footprint of conoid and trapezoid ligament in order to improve vertical stability of the AC joint. This method is based on CC ligament augmentation with a double flip button/polydioxanone (PDS), combined with V-loop pulley suture for anatomical fixation.

This is a prospective case report. Two professional, male basketball players in this study with a mean age of 25 years underwent surgery in 2019. Clinical subjective outcome, VAS score, Nottingham Clavicle Score, and radiological CC distance were measured before and after the operation.

There were noticeable improvement in the patients' recovery after two years since the operation. We introduce TTDB technique as one of the open techniques for acute AC joint injuries in limited-resource hospital setting.

## Introduction

Acute Acromioclavicular (AC) joint injuries are common among active population. According to Sirin et al. [1], this type of injury is responsible for about 40% to 50% of shoulder injuries in many contact sports. The number of this incidence on male is also five times higher than that of females, with the highest incidence reported occurring on those aged 20 to 30 years old [1].

Many techniques have been introduced to address this acute AC injuries setting, and they principally aim to reconstruct CC ligaments. The MINAR (minimally invasive acromioclavicular joint reconstruction) technique as explained by Rosslenbroich et al. is one of famous techniques widely used by many surgeons today [2]. This technique can be performed by a single tunnel-adjustable loop length suspensory fixation device. However, this technique requires specific medical tools that hospitals with limited resources do not have. Then, some loss of reduction become some issues with this original method as the two native components (trapezoid and conoid) of the original CC ligament are not restored. Breur et al. introduced triple button technique with double tunnel at clavicle. However, some loss of reduction still become some issues during follow-up [3]. In this paper, we introduce the TTDB technique to minimize this complication.

## Patients and method

This study involved two patients with acute Type V AC joint injuries. Both patients were 25 years old males, professional basketball players suffering from sport injuries in competition. After consenting to participate in this research, we performed the TTDB surgery technique. After two years since the operation, we measured clinical subjective outcome, VAS score, Nottingham clavicle score, and radiological CC distance.

The operations were performed under general anesthesia. The patients were treated in supine position with sandbag at upperback. Before the operation, one dose of prophylactic antibiotic was administered. An anterior deltopectoral approach was utilized with saber incision. The AC joint, the lateral end of the clavicle, and the coracoid process were exposed. Detachment of the deltotrapezial fascia from the clavicle subperiosteally was performed. Then, a drill hole was made into the coracoid base and a total of 3 holes in the lateral clavicle using a 2.2 mm drill bit, started 15 mm from the AC joint with a distance of 15 mm from each other. In our modified technique, two flip-buttons (Fliptack®, Karl Storz) were augmented with non-absorbable sutures, and a zip loop construct was created. One of the buttons can be carried through the coracoid and the remaining buttons through the center hole of the clavicle. The reduction was then performed. Then, we added two non-absorbable suture (Ultrabraid no.2, Smith Nephew) from the flip button at the coracoid through the two sides holes of the clavicle at the edge border of the footprint, both conoid and trapezoid ligament. As a result, there were two divergent force vectors reinforce of one vertical vector from previous double flip button loop. The deltotrapezial fascia was sutured, and then the wound was closed.

## Results

**Fig. 1:**
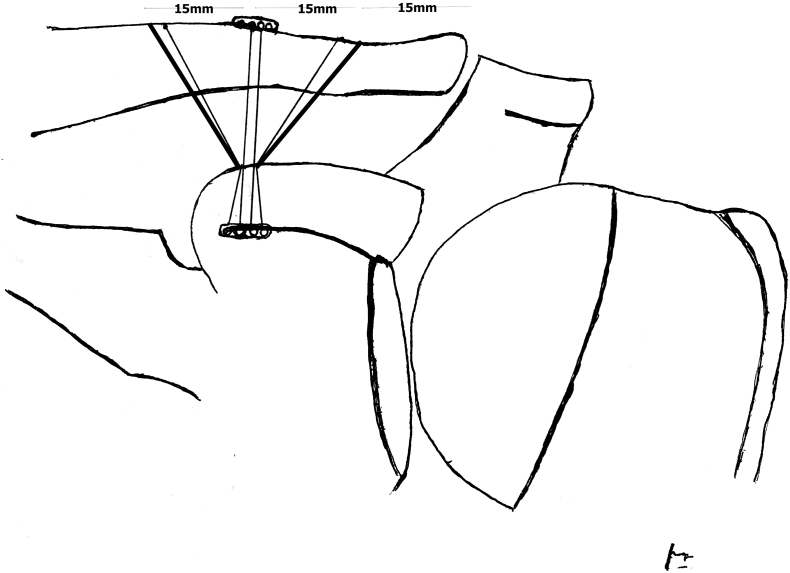
TTDB technique configuration.

**Fig. 2:**
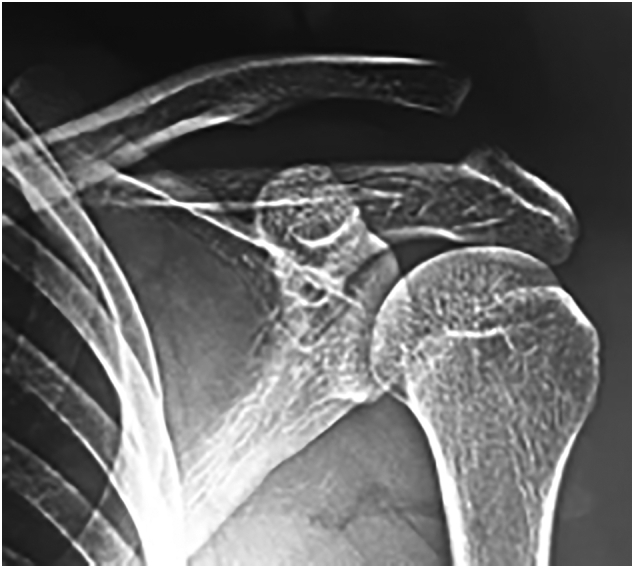
Pre operative X ray. AC joint dislocation Rockwood type V.

**Fig. 3:**
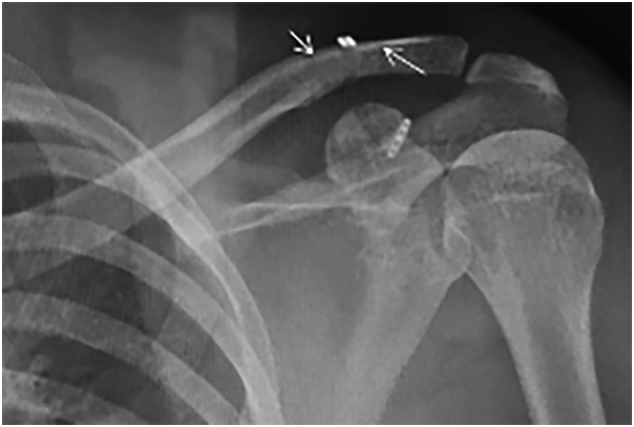
Post operative X ray. TTDB inserting site was marked with white arrow.

The results of X-ray scan before and after the operation is shown in Fig. 1, Fig. 2, Fig. 3.

The patients were immobilized with an arm sling bandage for four weeks after operation. After the four weeks, the ROM could be increased in line with the pain threshold, and then carried out without resistance for 12 weeks. Overhead work should be held off for three months and contact sports for up to six months.

The mean time from injury to surgery was two days, and the follow-up period was two years. There was no subjective complaint and any restriction of movement advanced at the patient's shoulder in the final follow up. VAS score decreased from 7 before the operation to 0 after the operation. Similarly, CC distance reduced from 18 mm to 8 mm. Meanwhile, Nottingham clavicle score increased from 34 to 98. In conclusion, no significant difference was detected between the last follow-up CC distance and that of the contralateral shoulder. All patients were back to work after a mean of ten weeks (range: 8–12 weeks), and returned to sport after about six months.

## Discussion

We introduce TTDB technique to acute AC joint injuries type V Rockwood classification. This method is based on CC ligament augmentation with double flip button/polydioxanone (PDS), typically used for extracortical ACL graft fixation, combined with the triple tunnel at clavicle with V-shaped suture at the edge border of footprint, both conoid and trapezoid ligament, for anatomical fixation. We added triple tunnel and double V-shaped suture in our technique to expand coverage of footprint of conoid and trapezoid ligament so that it increases anatomical fixation coverage and improve vertical stability of the AC joint. From the anatomical study, the average AC-joint distance to the medial end of its capsule was 0.7 cm (0.4–0.9) cm in females and 0.8 (0.4–1.2) cm in males. In females, the trapezoid ligament began, on average, at 0.9 (0.4–1.6) cm and ended at 2.4 (2.0–2.8) cm; for males, it began at 1.1 (0.8–1.6) cm and ended at 2.9 (2.1–3.8) cm. For the conoid ligament, the corresponding figures were 2.6 (2.0–3.7) cm and 4.7 (3.9–6.2) cm. [4]. We made three tunnels at the lateral clavicle, started 15 mm from AC joint with a distance of 15 mm from each other, mimicking the anatomy of CC ligament complex. To prevent a fracture of the clavicle, we used a small 2.2 mm drill bit size.

Many surgery techniques were introduced to treat acute AC injuries – more than 100 techniques – by creating single and double tunnels at the clavicle [3]. Rosslenbroich et al. introduced MINAR (minimally invasive acromioclavicular joint reconstruction) widely used by many surgeons [2]. Breur et al. modified this technique with the double tunnel at the clavicle by using triple buttons. Some loss of reduction still become an issue during the follow-up [3]. Moreover, some biomechanical studies showed that there was no difference in bidirectional strength and stability between the single-and double-clavicle tunnel techniques of coracoclavicular reconstruction.

The best surgical technique for AC joint dislocation is still in debate [5]. However, to our best knowledge, it is the first analysis of the triple tunnel with a double button at the clavicle in current literature. By using triple tunnels with smaller holes size, we mimic the anatomy of the CC ligament and expand the coverage of the CC ligament complex to improve vertical stability. We did not find any other surgical complications such as a fracture of clavicle, instability, prolonged pain, nerve damage, injury of blood vessels, or infection in our patients.

However, several aspects must be considered in choosing this technique. We introduce this technique for selected patients who (1) have acute injury, (2) are young, (3) have no concomitant injury.

Nevertheless, It must be taken into account that the sample size was too limited to give a final declaration.. Further biomechanical and clinical studies are necessary to prove improving vertical instability after performing this technique.

## Funding

This research received no external funding.

## Declaration of competing interest

The authors declare no conflict of interest.
